# IL-17 Triggers Invasive and Migratory Properties in Human MSCs, while IFNy Favors their Immunosuppressive Capabilities: Implications for the “Licensing” Process

**DOI:** 10.1007/s12015-020-10051-4

**Published:** 2020-10-16

**Authors:** Bárbara Du-Rocher, Renata Binato, Julio Cesar Madureira de-Freitas-Junior, Stephany Corrêa, André Luiz Mencalha, José Andrés Morgado-Díaz, Eliana Abdelhay

**Affiliations:** 1 Stem Cell Laboratory, Bone Marrow Transplantation Center, National Cancer Institute, Rio de Janeiro, Brazil; 2 Present Address: Laboratory on Thymus Research, Oswaldo Cruz Institute, Oswaldo Cruz Foundation, Rio de Janeiro, Brazil; 3 Cellular and Molecular Oncobiology Program, National Cancer Institute, Rio de Janeiro, Brazil; 4 Laboratory of Cancer Biology, Biophysics and Biometry Institute, University of State of Rio de Janeiro, Rio de Janeiro, Brazil

**Keywords:** Mesenchymal stromal cells (MSCs), IFNy, IL-17, Chip array, Immunomodulation

## Abstract

Mesenchymal stromal cells (MSCs) were first used as a source for cell therapy in 1995; however, despite their versatility and unambiguous demonstration of efficacy and safety in preclinical/phase I studies, the positive effect of MSCs in human phase III studies did not resemble the success obtained in mouse models of disease. This dissonance highlights the need to more thoroughly study the immunobiology of MSCs to make better use of these cells. Thus, we aimed to study the immunobiology of MSCs by using chip array analysis as a method for general screening to obtain a global picture in our model study and found IFNy and IL-17 signaling as the first two “top canonical pathways” involved in MSCs immunomodulation. The role of IFNy in triggering the immunosuppressive properties of MSCs is well recognized by many groups; however, the role of IL-17 in this process remains uncertain. Interestingly, in contrast to IFNy, which actively improved the MSCs-mediated immunosuppression, IL-17 did not improve directly the MSCs-mediated immunosuppression. Instead, IL-17 signaling induced the migration of MSCs and inflammatory cells, bringing these cell types together and increasing the likelihood of the lymphocytes sensing the immunosuppressive molecules produced by the MSCs. These effects also correlated with high levels of cytokine/chemokine production and metalloprotease activation by MSCs. Importantly, this treatment maintained the MSCs safety profile by not inducing the expression of molecules related to antigen presentation. In this way, our findings highlight the possibility of using IL-17, in combination with IFNy, to prime MSCs for cell therapy to improve their biological properties and thus their therapeutic efficacy. Finally, the use of preactivated MSCs may also minimize variations among MSCs to produce more uniform therapeutic products. In the not-so-distant future, we envisage a portfolio of MSCs activated by different cocktails specifically designed to target and treat specific diseases.

**Electronic supplementary material:**

The online version of this article (10.1007/s12015-020-10051-4) contains supplementary material, which is available to authorized users.

**Figure:**
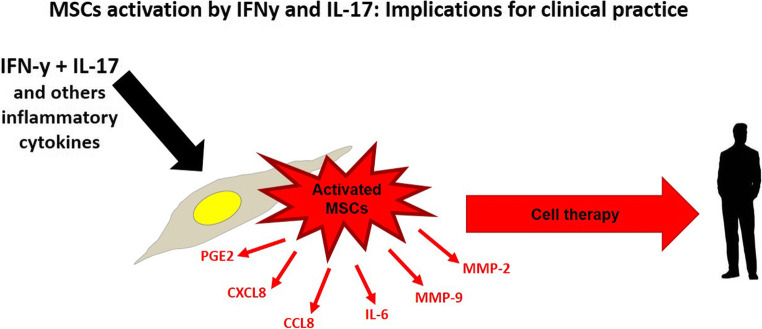
Graphical abstract

## Introduction

Mesenchymal stromal cells (MSCs) were first used as source for cell therapy in 1995, and since then, they have become one of the most widely used cell types in preclinical studies and clinical trials, reaching the impressive number of 9447 clinical trials registered so far at the clinicaltrials.gov platform (https://clinicaltrials.gov/, term: MSCs, September/2020). The main reason why these cells have become so attractive is their ability to produce molecules capable of inducing tissue regeneration and inhibiting undesirable immunological responses. Associated with these effect, the potential of MSCs to differentiate into at least three distinct subtypes of cells (osteoblasts, chondroblasts and adipoblasts) and their relative ease of isolation, culture and expansion also encourage studies and physicians to make use of these cells [1]. Additionally, because MSCs seem to be relatively safe, due to their low capacity for transformation and low immunogenicity profile (low expression of MHC I and II and no expression of costimulatory molecules), they are considered a promising tool for cellular therapy of degenerative and immune diseases [2, 3]. Altogether, these unique characteristics made MSCs the ideal cells for cell therapy by rendering their application in clinic much more feasible than that of other cell therapies [4]. However, it has been more than 20 years since their first use, and despite their versatility and unambiguous demonstration of efficacy and safety in preclinical/phase I studies, MSCs have not achieved therapeutic effects in human phase III studies that resemble the success obtained in mouse models of disease. In fact, only three recent phase III randomized studies have shown significant efficacy for MSCs relative to a placebo: Allogeneic Marrow MSCs for GvHD (NCT02336230), Autologous Marrow MSCs for Heart Failure (NCT01768702) and Adipose MSCs for Enterocutaneous Fistular Disease (NCT01541579). Interesting, all of these studies were adaptive clinical trials designed from their respective original unsuccessful trials that did not meet their primary endpoints [4, 5] . Unfortunately, all other trials, i.e., the majority of studies performed to date, have failed, leading to questions regarding why such differences between preclinical and phase III studies occur. This discrepancy is generally attributed to differences in MSCs isolation, manipulation and administration, such as the donor source (autologous vs. allogeneic), tissue origin (bone marrow is the most common), route of administration (systemic vs. local), dosing (number of MSCs/dose and number of doses) and MSCs culture status (fresh vs. cryopreserved/thawed) [1] . Eligibility criteria and primary endpoints for clinical trials also contribute to the conflicting results observed among clinical trials [3].

As the mechanism of action of MSCs is not fully elucidated, we still have the opportunity to discover new approaches to stimulate the therapeutic potential of these cells. In this way, because IFNy has long been known to be involved in priming MSCs for immunosuppression, pretreatment of MSCs was suggested by Guess. Importantly, the investigators reported a good manufacturing practice-compliant MSCs manufacturing protocol to generate IFNy-primed MSCs while maintaining the safety profile of the MSCs [3]. Here, using chip array analysis, we found the IL-17 signaling pathway to be the second most important pathway in MSCs activation, highlighting the possibility of using IL-17, in combination with IFNy, to prime MSCs for cell therapy in order to improve their therapeutic efficacy.

## Material and Methods

### Cell Culture

Bone marrow and peripheral blood samples were obtained from healthy donors after written informed consent. This work was approved by the local ethics committee of Instituto Nacional de Câncer (Rio de Janeiro, Brazil, protocol number: #034/06).

#### MSCs Primary Culture and Expansion

MSCs were isolated from heparinized bone marrow samples. Briefly, mononuclear cells were obtained by density-gradient centrifugation (Histopaque 1.077 g/mL; Sigma–Aldrich, St. Louis, USA) and seeded at 2 × 10^5^ cells/cm^2^ in MSCs complete medium, consisting of DMEM - low glucose (DMEM-LG, Invitrogen, Carlsbad, USA), supplemented with 10% fetal bovine serum (FBS; HyClone, Waltham, USA), 2 mM glutamine (Invitrogen) and 100 U/mL penicillin with 100 μg/mL streptomycin (Sigma–Aldrich). Cells were incubated at 37 °C in a humidified 5% CO_2_ atmosphere, allowed to adhere for 48 h, and then washed with PBS to remove the non-adherent cells (This cultures were called Passage 0). Half of the medium was changed twice a week until 80–90% cell confluence was reached. After that, cells were trypsinized (0.1% trypsin; Invitrogen) at 37 °C for 3 min and reseeded at 4 × 10^3^ cells/cm^2^. In order to have a less heterogeneous population of MSCs, we used only cultures after the second passage. These cells met the minimal criteria for defining multipotent MSCs, as defined by The International Society for Cellular Therapy [6] since they were plastic-adherent cells able to differentiate into adipocytes, osteoblasts and chondrocytes and expressed CD73, CD90, and CD105 in the absence of lineage commitment markers, such as CD14, CD19, CD34, CD45, and HLA-DR [7].

#### MSCs Treatment with IFNy, IL-17 or Both

MSCs primary cultures at less than 80–90% confluence (a cell density of approximately 12,000 cells/cm^2^) were incubated for 3 days in the absence or presence of IFNy (10 ng/ml), IL-17 (10 ng/ml ➔ 5 ng/ml IL-17A + 5 ng/ml IL-17F) or both IL-17 and IFNy (10 ng/ml for each), and the supernatant was collected, centrifuged to isolate the cell-free medium and frozen. Alternatively, MSCs were incubated for 24 h in the absence or presence of these cytokines and then washed twice with PBS and directly used in a lymphocyte proliferation assay or trypsinized (0.1% trypsin; Invitrogen) at 37 °C for 3 min for other assays. The recombinant cytokines were purchased from R&D Systems (Minneapolis, USA).

#### Lymphocyte Proliferation

Peripheral blood mononuclear cells (PBMCs) were isolated from heparinized blood samples from healthy donors by density-gradient centrifugation (Sigma–Aldrich) and labeled with 3 μM CFSE (Invitrogen) according to the manufacturer’s protocols. PBMCs were then stimulated with allogeneic PBMCs in a one-way mixed leukocyte reaction (MLR) by incubating 5 × 10^5^ PBMC responder cells with 5 × 10^5^ irradiated (2500 cGy) PBMC unrelated stimulator cells in a final volume of 1 mL/well in 24-well flat-bottomed tissue-culture plates for 7 days. Alternatively, PBMCs were stimulated with the polyclonal stimulus anti-CD3/CD28 (12,5 μl/ml, Dynabeads Human T-Activators CD3/C28, Life Technologies, Carlsbad, USA) + recombinant IL-2 (30 ng/ml, BioLegend, San Diego, USA) for 3 days. To test the effect of MSCs on lymphocyte proliferation, 5 × 10^4^ third-party MSCs (10% MSCs, relative to the PBMC responder cells) were added per well as the gold standard; however, other concentrations were also used and are specified. A transwell insert membrane with a 0.4-μm pore size (Corning, New York, USA) was used in some experiments to prevent cell-cell contact, and MSCs were seeded in the lower chamber. Other modifications were the use of: 1. A transwell insert membrane with an 8-μm pore size (Corning), allowing lymphocyte migration, 2. Supernatant samples from MSCs (previously treated for 3 days with or without IFNy, IL-17 or both, as described above), or 3. The use of MSCs (previously treated for 24 h with or without IFNy, IL-17 or both, as described above). To test if MSCs are immunogenic or not, MSCs were also used as stimulators cells for lymphocyte proliferation. In this case, 5 × 10^5^ PBMC responder cells were stimulated with 5 × 10^5^ MSCs (previously treated for 24 h with or without IFNy, IL-17 or both, as described above) + recombinant IL-2 (30 ng/ml, BioLegend) in a final volume of 1 mL/well in 24-well flat-bottomed tissue-culture plates and incubated for 3 days. Cultures were maintained in RPMI-1640 medium (Invitrogen) supplemented with 10% FBS (HyClone), 2 mM glutamine (Invitrogen) and 100 U/mL penicillin with 100 μg/mL streptomycin (Sigma–Aldrich), incubated at 37 °C in a humidified 5% CO_2_ atmosphere and further analyzed by flow cytometry.

### Expression Chip Array

To performed chip array assay, total RNA was isolated from four different samples using the RNeasy Mini Kit (Qiagen, Düsseldorf, Germany): 1. MSCs cultured alone; 2. an MLR cultured alone and 3–4. MSCs and an MLR cocultured but separated by a transwell membrane with a 0.4-μm pore size (Corning) after 3 days of incubation. The RNA extraction was carried out according to the manufacturer’s protocols and them 100 ng of total RNA was used to synthesize biotinylated cRNA using the GeneChip Whole Transcription (WT) Sense Target Labeling Assay Kit (Affymetrix, Santa Clara, USA). This biotinylated cRNA and subsequently hybridized to GeneChip Human Exon 1.0 ST Arrays (Affymetrix), washed and stained according to the manufacturer’s protocols. The arrays were scanned using a GeneChip® Scanner 3000 and, thereafter, Affymetrix Expression Console software version 1.0 was used to create summarized expression values (CHP-files). Partek® software (http://www.partek.com) was used to analyzed the data, and a fold change ≥5 was used as a criteria to determine differentially expression genes exhibiting overexpression or downregulation. Pathway analysis and related processes were performed using Ingenuity Pathway Analysis™ (IPA) software (https://www.qiagenbioinformatics.com/products/ingenuity-pathway-analysis/) and the databases NCBI gene (https://www.ncbi.nlm.nih.gov/gene) and GeneCards V3 (https://www.genecards.org/).

### Real-Time Quantitative Polymerase Chain Reaction

MSCs were incubated alone (5 × 10^4^ cells/well in a 24-well plate) or in the presence of an MLR in the upper chamber of a transwell membrane with a 0.4-μm pore size (Corning) for up to 4 days. Total RNA from MSCs was obtained with TRIzol LS reagent (Invitrogen) according to manufacturer’s protocol and stored at −70 °C until use. Real time quantitative PCR (RT-qPCR) was applied for mRNA transcripts quantification. For this expression analysis, 2 μg RNA was reverse transcribed with Superscript II Reverse Transcriptase® (Invitrogen). The mixes contained cDNA dilutions (1:100), 50% of SYBR Green PCR Master Mix® (Applied Biosystems, Carlsbad, USA) and primers for the genes of interest. The reaction consisted of 50 cycles of 20 s at 95 °C, 30 s at 60 °C and 30 s at 72 °C, and was performed on a Rotor Gene 6000 thermocycler (Corbett, Sydney, Australia). Normalization was carried out with GAPDH mRNA levels. Relative expression was calculated using the delta delta cycle threshold (DDCt) method. The following primers were used: COX-2: forward primer, 5′ -CAG ACG CCC TCA GAC AGC AAA- 3′ and reverse primer, 5′ -ATG GGT GGG AAC AGC AAG GAT- 3′; CCL8: forward primer, 5′ -CTC AGG GAC TTG CTC AGC C- 3′ and reverse primer, 5′ -CCT CCT TGC CCC GTT TG- 3′; CXCL8: forward primer, 5′ -GGT GCA GAG GGT TGT GGA GAA G- 3′ and reverse primer, 5′ -ACC CTA CAA CAG ACC CAC ACA A- 3′ and GAPDH: forward primer: 5′ -ACCACAGTCCATGCCATCAC- 3′, reverse primer: 5′ -CCACCACCCTGTTGCTGTA- 3′.

### Flow Cytometry

All flow cytometry experiments were analyzed by using the protocols described below. Data were acquired using a FACSCalibur flow cytometer and analyzed using Paint a Gate or CellQuest software (BD Biosciences, San Jose, USA).

#### Protein Expression by Cell-Surface and Intracellular Staining

MSCs were processed into single-cell suspensions by trypsin digestion (0.1% trypsin; Invitrogen). For cell-surface staining, cells were labeled with antibodies according to the manufacturer’s protocol, followed by fixation in PBS containing 1% paraformaldehyde (PBS/PFA 1%). The antibodies used were as follows: anti-MHC I (clone G46–2.6, BD Pharmingen, San Jose, USA), anti-MHC II (clone L243, BD Pharmingen), anti-IL-17RA (clone 133,617, R&D Systems), anti-IL-17RC (clone 309,822, R&D Systems), anti-CD80 (clone L307.4, BD Pharmingen), anti-CD86 (clone 2331-FUN-1, BD Pharmingen), anti-CD40 (clone 5C3, BD Pharmingen) and corresponding isotype controls. For intracellular staining, cells were fixed with PBS/PFA (0,4%), permeabilized with PBS/BSA (1%)/Tween 20 (0,5%) and labeled with antibodies according to the manufacturer’s protocol. The antibodies used were as follows: anti-CXCL8 (clone: G265–8, BD Pharmingen), anti-CCL8 (clone C-17, Santa Cruz Biotechnology, Dallas, USA), anti-COX2 (polyclonal Goat IgG, R&D Systems), anti-Act-1 (clone: WW-18 + a secondary IgG2a-FITC antibody, both from Santa Cruz Biotechnology) and corresponding isotype controls. Twenty thousand events were collected for each sample. The results were expressed as the percentage of positive cells or as the median relative fluorescence intensity (MRFI), calculated by subtracting the median fluorescence intensity (MFI) of the isotype control from the MFI of the corresponding specific antibody.

#### Lymphocyte Proliferation by Carboxyfluorescein Diacetate Succinidyl Ester (CFSE) Detection

PBMCs from lymphocyte proliferation assays were harvested, washed in PBS, resuspended in PBS/BSA (1%) and immediately acquired. Twenty thousand CFSE+ events were collected for each sample. The results were expressed as the percentage of cells positive for CFSE (hi or low).

#### Cell Cycle Distribution by Propidium Iodide (PI) Staining

MSCs were harvested, washed with PBS, reconstituted in a hypotonic buffer (0.1% sodium citrate, 0.1% Triton-X, 100 μg/mL RNase and 50 μg/mL PI) and immediately acquired. Ten thousand events were collected for each sample. The results were expressed as the percentage of cells in each cell cycle phase. Results were expressed as percentage of cells in G0/G1, S and G2/M phases.

### Enzyme-Linked Immunosorbent Assay (ELISA)

IL-6 and IL-8 levels in the supernatants of cultures of an MLR and MSCs were determined using antibody-specific ELISA kits (PeproTech, Rocky Hill, USA) with internal controls according to the manufacturer’s instructions. The experiments were performed in triplicate and analyzed using an ELISA microplate reader at 450 nm Asys Expert Plus (Biochrom, Holliston, USA).

### Migration/Invasion Assays

MSCs (3 × 10^4^) were seeded on transwell membrane with an 8-μm pore size coated with Matrigel (BD Biosciences). Medium containing 10% FBS was added as a chemoattractant in the lower chamber, and after 3 days of incubation, the upper surface of the membrane was scrubbed with a swab. The cells were fixed with ethanol and stained with crystal violet. The visualization of the stained cells was performed using an Axio Observer Z1 microscope (Zeiss, Oberkochen, Germany).

### Zymography Assay

Equal amounts of protein from the conditioned culture medium of MSCs that received different treatments were subjected to SDS-PAGE containing 0.2% gelatin. After electrophoresis, the gels were washed twice in 10 mM Tris/ HCl (pH 8.8) containing 2.5% Triton X-100 and then incubated in an activation buffer (0.02% NaN3, 5 mM CaCl2, and 50 mM Tris/HCl, pH 8.0) at 37 °C overnight. The gels were then stained with Coomassie Brilliant Blue and destained in a solution containing 40% methanol and 10% acetic acid. The gelatinolytic activity of the MMPs was detected as clear bands on the blue background.

### Statistical Analysis

Student’s t test or one-way analysis of variance (ANOVA) was performed and *P*-values less than 0.05 were considered statistically significant (*p < 0.05, **p < 0.01, ***p < 0.001 and ****p < 0,0001). Statistical analyses and graphing were performed using GraphPad Prism 7® software (GraphPad, La Jolla, USA).

## Results

### MSCs Increase the Levels of Molecules Associated with Immunological Response After Activation by an MLR in a Contact-Independent Manner

**Fig.1:**
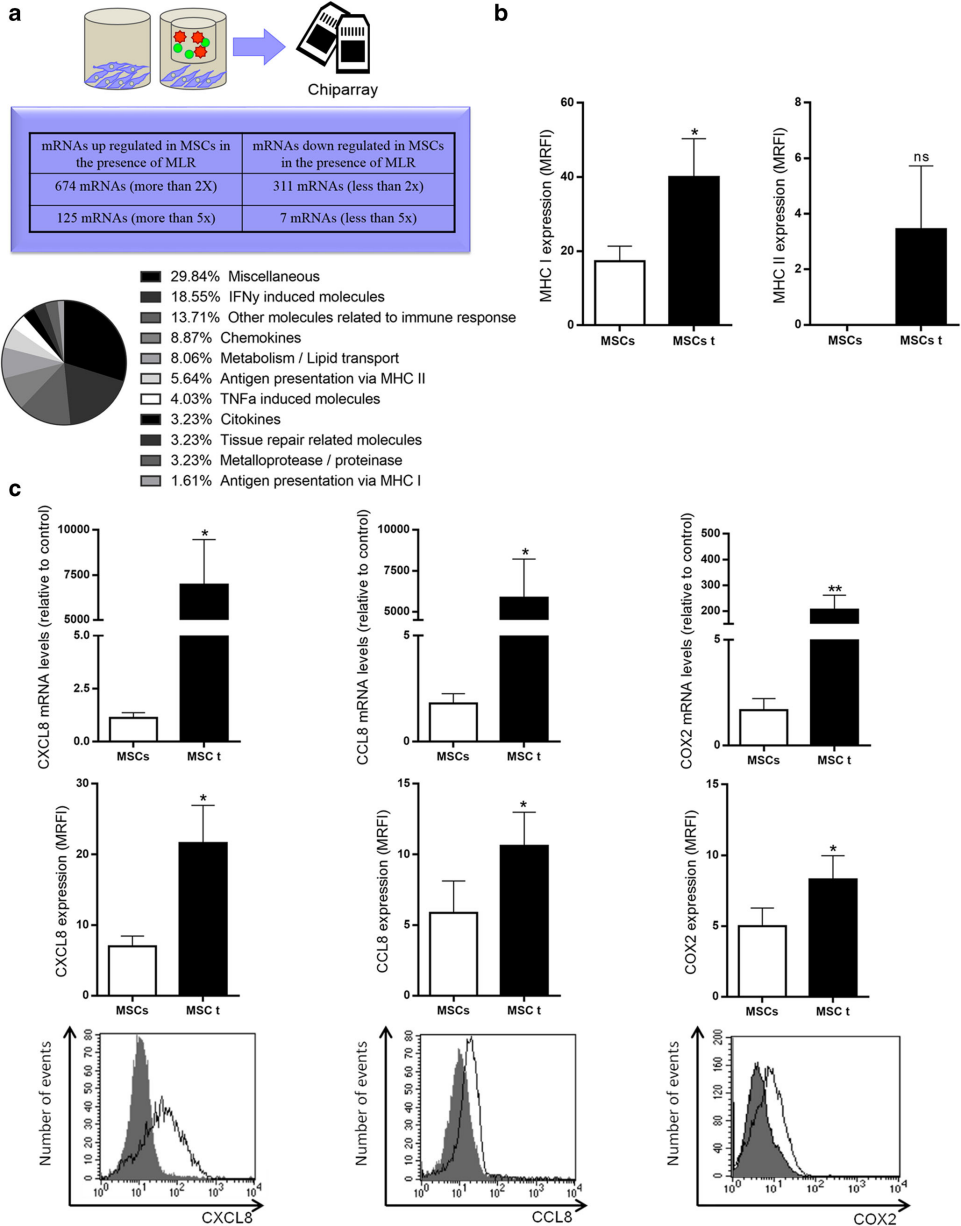
**MSCs cocultured with an MLR possess an activated phenotype rich in molecules associated with the immunological process.** MSCs populations were analyzed after 3 days of culture in the absence (MSCs) or presence (MSCs t) of MLRs separated by a transwell membrane. **a** Total numbers of mRNA transcripts up- or downregulated 2- or 5-fold in the MSCs t, and a pie chart summarizing the average frequencies of major functions and/or families of mRNAs upregulated more than 5-fold in the MSCs t. The categorization of clusters was performed based on information provided by GeneCards V3 and NCBI gene. **b** MHC class I and II protein expression by flow cytometry. **c** CXCL8, CCL8 and COX-2 gene and protein expression by real time PCR and flow cytometry (respectively), and a representative assay showing overlaid histograms for protein expression (black line – MSCs t; gray shadow – MSCs; isotype controls are not shown). Results are expressed as the mean ± SEM of four independent experiments (**b**) and five to eleven independent experiments (**c**). *p<0,05; **p<0,01; ns – not significant. MRFI: median relative fluorescence intensity

To identify the immunosuppressive mechanism exerted by MSCs, we performed chip array experiments as a method for general screening to obtain a global picture in our model study. For this approach, MSCs were cultured in the absence or presence of an MLR, separated by a transwell membrane (0,4 μm), and total mRNA was extracted from the MSCs alone (steady-state inactive MSCs) and MSCs t (cocultured with the MLR). We used the GeneChip whole transcript – WT array (Affymetrix) containing all the mRNAs expressed by the human genome (27,000) as a chip array tool to analyze our data and found 674 mRNAs that exhibited at least a 2-fold increase in expression in the MSCs t compared to the steady-state inactive MSCs. Furthermore, the MSCs activated by the MLR exhibited at least a 2-fold decrease in the mRNAs levels of 311 genes (Fig. 1a). In searching for molecules that are potentially important in triggering the immunosuppression exerted by MSCs, we looked at genes whose mRNA levels were altered by at least 5-fold and found 125 mRNAs with increased expression and 7 mRNAs with decreased expression (SuplTable 1). Seventy percent of all the genes upregulated at least 5-fold were somehow involved in immunosuppression, and among them, we observed many genes involved in MHC class I and II presentation (~7%), chemokine genes (~9%), genes involved in lipid metabolism and transport (~8%) and genes encoding regulatory proteins induced by IFNy (~18%) (Fig. 1a, b). For chip array validation we chose to evaluate the mRNA levels of the molecules CXCL8 (165-fold), CCL8 (101-fold) and COX-2 (47-fold). Our real-time PCR results demonstrated higher expression of the CXCL8, CCL8 and COX-2 transcripts in MSCs cocultured with an MLR than in MSCs cultured under steady-state conditions (Fig. 1c). This induction was time dependent (SuplFig 1A) and correlated with protein levels (Fig. 1c). Moreover, these MSCs were able to inhibit lymphocyte proliferation and their immunosuppressive properties were inhibited by indomethacin, a nonselective COX inhibitor (SuplFig 1B).

### MSCs Increase the Expression of Many Genes Involved in the IFNy and IL-17 Pathways, Possess the Necessary Apparatus to Respond to IL-17 and Increase their Cellular Activities Related to Migration/Invasion After Activation by an MLR

**Fig. 2:**
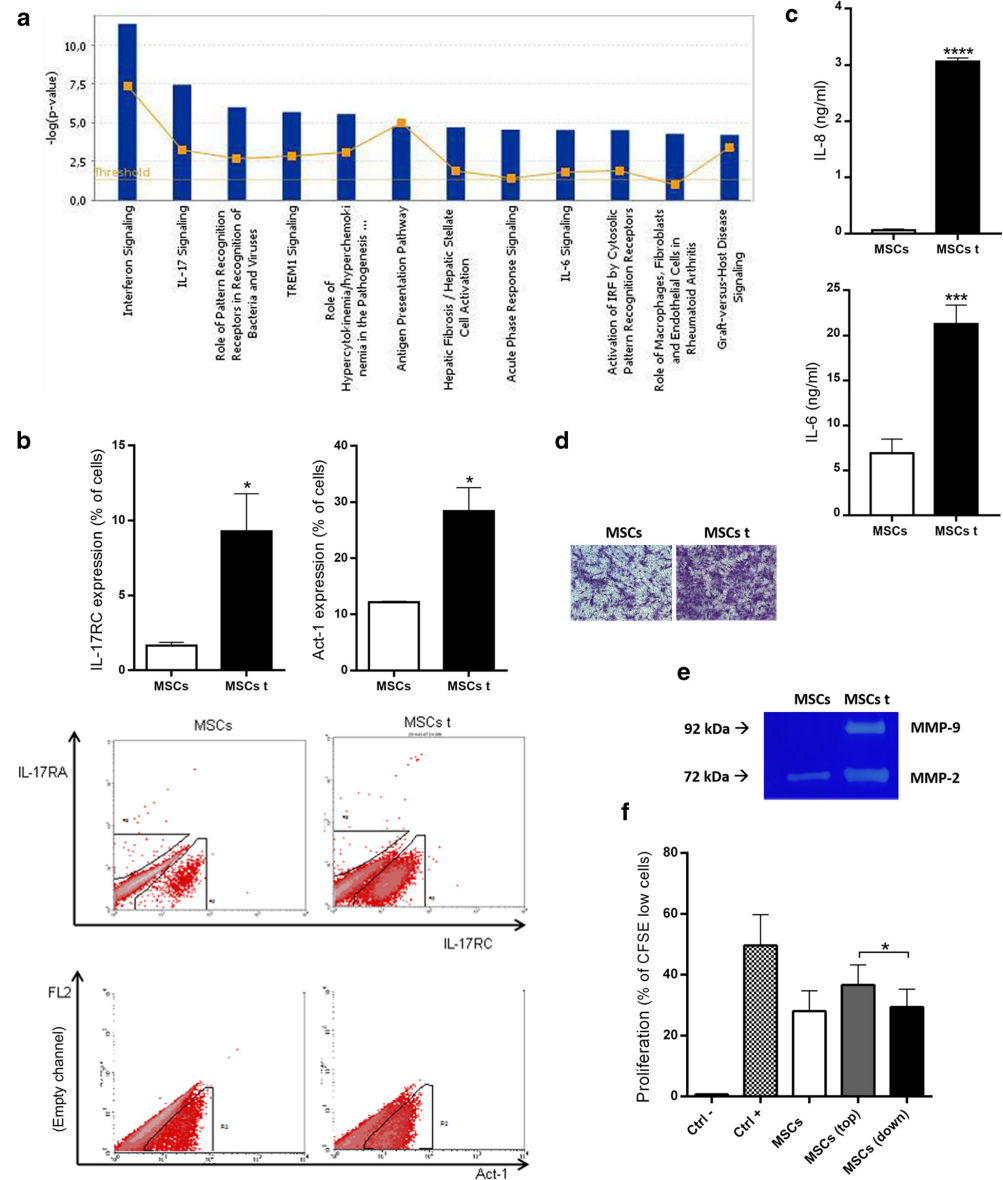
**MSCs cocultured with an MLR upregulate the expression of many genes and proteins involved in IL-17 signaling and increase cellular activities related to migration/invasion.** MSCs populations were analyzed after culture in the absence (MSCs) or presence (MSCs t) of MLRs separated by a transwell membrane. **a** Ingenuity Systems analysis showing the top canonical pathways activated in the MSCs t by the MLR after 3 days of incubation. **b** IL-17RC and Act-1 protein expression by flow cytometry after 3 days of incubation, and dot plots of a representative assay. **c** IL-8 and IL-6 secretion by ELISA after 3 days of incubation. **d** Invasion/migration through matrigel + transwell membrane after 5 days of incubation. **e** Metalloprotease activity by zymography assay after 5 days of incubation. **f** Lymphocyte proliferation by CFSE detection (flow cytometry) after 7 days of incubation. Ctrl- (Negative control, PBMC without stimulus), Ctrl+ (Positive control, MLR), and test groups: MSCs, MSCs (top) - Lymphocytes that did not migrate closer to MSCs and MSCs (down) - Lymphocytes that did migrate closer to MSCs. Results are expressed as the mean ± SEM of six independent experiments (**b**), four to five independent experiments (**c**), three independent experiments (**d**), four independent experiments (**e**) and ten independent experiments (**f**). *p<0,05; ***p<0,001; ****p<0,0001

Using the software Ingenuity Pathway Analysis (IPA), we clustered the mRNAs with increased expression into common biological pathways, generating a score based on the corresponding *p* value. The higher the score, the more likely that the pathway was involved in our biological hypothesis. In this way, IFNy signaling and IL-17 signaling were ranked as the first two “top canonical pathways” based on only the mRNAs that were upregulated at least 5-fold (Fig. 2a). Because of this finding, we first analyzed the major IL-17 subunit receptors (IL-17RA and IL-17RC) and Act-1, the immediately downstream molecule in IL-17 signaling, in MSCs. Under steady-state conditions, MSCs express IL-17RC and Act-1 but not IL-17RA (Fig. 2b). Moreover, IL-17RC and Act-1 levels were increased in MSCs after activation by an MLR (Fig. 2b) as well as IL-6 and IL-8 (Fig. 2c).

Using an invasion assay, we observed that MSCs activated by an MLR had largely increased migratory and invasive properties, and this effect was accompanied by large increases in MMP-2 and MMP-9 activity (Fig. 2d, e). We also performed an MLR in the upper chamber of an 8-μm transwell insert in the presence or absence of MSCs in the lower compartment. As shown in Fig. 2f, the lymphocytes that migrated through the 8-μm transwell membrane (MSCs down), reaching the lower compartment, proliferated less than the lymphocytes that remained in the upper compartment (MSCs top) (Fig. 2f). Still performing our model characterization, we observed that MSCs activated by an MLR proliferated more than steady-state MSCs (SuplFig 3).

### IL-17 Triggers the Invasive and Migratory Properties of MSCs, While IFNy Favors their Immunosuppressive Capabilities

**Fig. 3:**
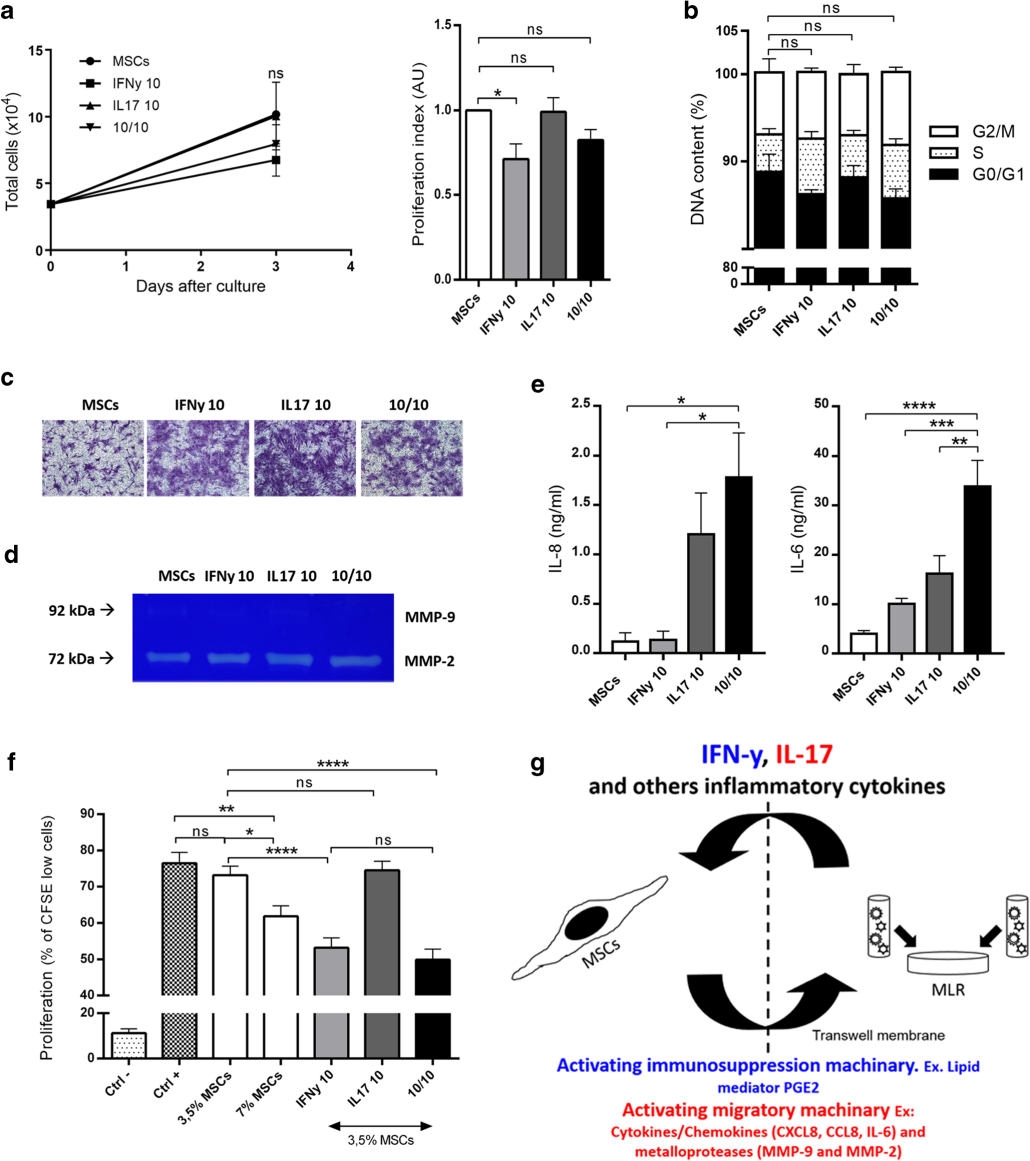
**IL-17 triggers invasive and migratory properties in MSCs, while IFNy favors the immunosuppressive capabilities of these cells.** MSCs populations were analyzed after 3 days of culture in the absence (MSCs)or presence of IFNy (IFNy 10), IL-17 (IL17 10), or both IL-17 and IFNy (10/10). **a** Proliferation by cell counts. **b** Cell cycle by PI staining (flow cytometry). **c** Invasion/migration through matrigel + transwell membrane. **d** Metalloprotease activity by zymography assay. **e** IL-8 and IL-6 secretion by ELISA. **f** Lymphocyte proliferation by CFSE detection (flow cytometry). Ctrl- (Negative control, PBMC without stimulus), Ctrl+ (Positive control, PBMCs + anti-CD3/CD28 + rIL-2), and test groups: 3,5% MSCs, 7% MSCs, IFNy 10 (3,5% MSCs pre-activated with IFNy), IL17 10 (3,5% MSCs pre-activated with IL-17) and 10/10 (3,5% MSCs pre-activated with IFNy and IL-17). MSCs were pre-activated for 24h. **g** Hypothesis proposed by our group. Results are expressed as the mean ± SEM of six independent experiments (**a**), four independent experiments (**b**), three independent experiments (**c**), three independent experiments (**d**), five to six independent experiments (**e**) and thirteen independent experiments (**f**). *p<0,05; **p<0,01; ***p<0,001; ****p<0,0001; ns – not significant

To elucidate the roles of IL-17 and IFNy, we treated MSCs with IL-17, IFNy or both (Doses defined by IL-6 and IL-8 production, SuplFig 4A). Treatment with these cytokines dont change MSCs morphology nor induce MSCs proliferation (SuplFig 4B and Fig. 3a, b). Regarding migration/invasion induction and metalloprotease activity in MSCs, treatment with IL-17 had the most pronounced effect, followed by IFNy. Interestingly, cotreatment did not result in a potentialization effect (Fig. 3c, d). IL-8 production was induced only by IL-17 or cotreatment, not by IFNy alone, while IL-6 was induced by IFNy and, to a lesser degree, IL-17 or cotreatment (Fig. 3e). Importantly, the effect of IL-17 was mediate by IL-17A, not by IL-17F (SuplFig 4C). In both cases, increased production of IL-8 and IL-6 was achieved by cotreatment. To test if cotreatment would have a synergistic effect on lymphocyte proliferation, MSCs were treated with IFNy, IL-17 or both for 72 h, washed to remove excess cytokines and used for a lymphocyte proliferation assay (performed on top of the MSCs). To better define changes in the suppressive capacity of treated MSCs, a suboptimal 3.5% MSCs dose (Dose defined by lymphocyte proliferation inhibition, SuplFig 4D) was used. Lymphocyte inhibition was dependent on the MSCs dose, and 3.5% IFNy-treated MSCs was more inhibitory than 7% untreated MSCs. IL-17 did not affect lymphocyte proliferation, and cotreatment had the same impact as IFNy treatment, so we did not observe any synergistic effect on lymphocyte proliferation inhibition (Fig. 3f). No effect was also observed when we used conditioned medium from treated MSCs instead of the MSCs (SuplFig 4E).

Figure 3g summarizes the mechanism of immunosuppression exerted by MSCs, which involves a dynamic process that requires constant cross-talk among MSCs, lymphocytes and other cells. This cross-talk involves MSCs activation by IFNy and IL-17 and the consequent production of cytokines/chemokines and metalloproteases (Fig. 3g).

### MSCs Activated by Inflammatory Cytokines do not Become Immunogenic

**Fig. 4:**
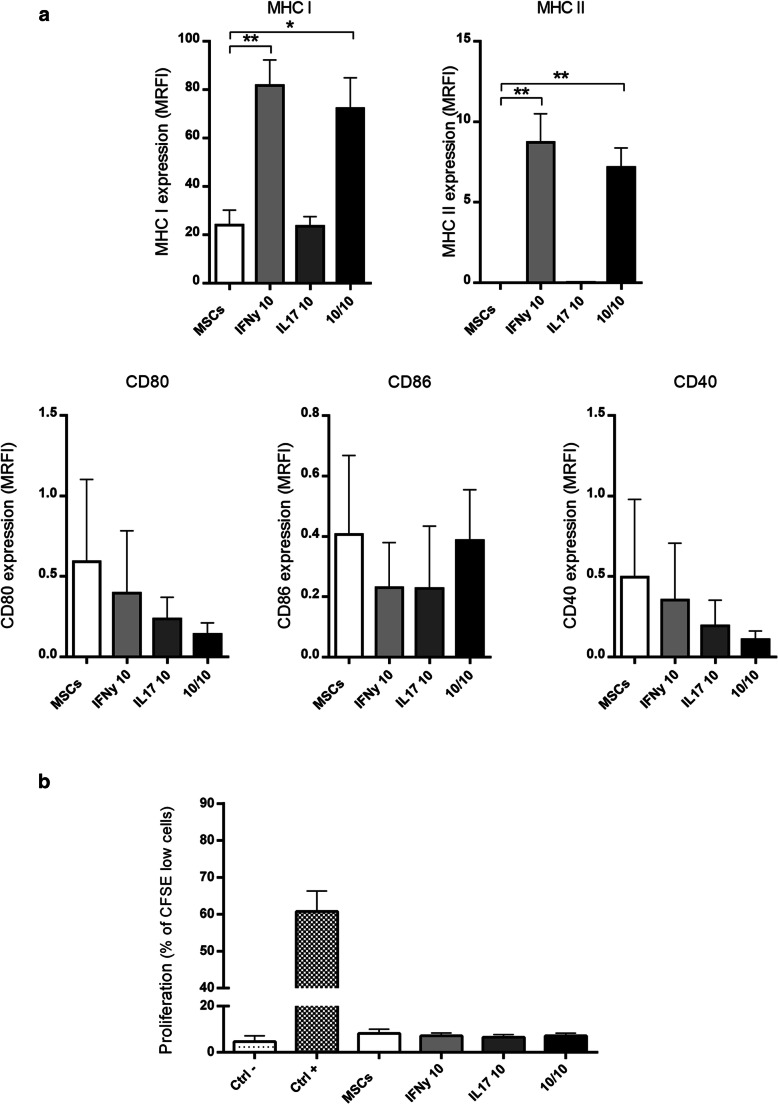
**MSCs activated by inflammatory cytokines do not became immunogenic.** MSCs populations were analyzed after 3 days of culture in the absence (MSCs)or presence of IFNy (IFNy 10), IL-17 (IL17 10), or both IL-17 and IFNy (10/10). **a** MHC I, MHC II, CD80, CD86 and CD40 protein expression by flow cytometry. **b** Lymphocyte proliferation by CFSE detection (flow cytometry). Ctrl- (Negative control, PBMC without stimulus), Ctrl+ (Positive control, PBMCs + anti-CD3/CD28 + rIL-2), and test groups: MSCs (PBMCs + MSCs + rIL-2), IFNy 10 (PBMCs + MSCs pre-activated with IFNy + rIL-2), IL17 10 (PBMCs + MSCs pre-activated with IL-17 + rIL-2), and 10/10 (PBMCs + MSCs pre-activated with IFNy + IL-17 + rIL-2). MSCs were pre- activated for 24h. Results are expressed as the mean ± SEM of three independent experiments (**a**) and six independent experiments (**b**). *p<0,05; **p<0,01. MRFI: median relative fluorescence intensity

The effects of IFNy, IL-17 and cotreatment on MHC class I and II and costimulatory molecule expression as well as their abilities to trigger lymphocyte proliferation under these specific conditions were analyzed. IFNy and cotreatment induced MHC class I and II expression but not costimulatory molecule expression, while IL-17 had no effect (Fig. 4a). Functionally, treated MSCs, even those treated with IFNy, were not able to induce lymphocyte proliferation (Fig. 4b).

## Discussion

Due to their versatility and promising therapeutic capabilities, MSCs have entered clinical application despite there being no clear definition of their mechanism of action, which may also account for the low success rate in the clinic. Here, we presented some results showing that the global mRNA expression of MSCs increased after coculture with MLR, suggesting that the cells switched from an inactive steady state to an activated phenotype. Among the mRNAs with increased expression, many of them such as IDO1, PDL, COX2, IL-6 and LIF (the expression of all of them increased by at least 12-fold) have already been demonstrated to be involved in the immunosuppression exerted by MSCs, corroborating our idea that the mechanism underlining MSC-mediated immunosuppression involves multiple molecules. The induction of genes involved in MHC class I and II presentation suggests that MSCs may, under certain conditions, present antigen and function as conditional APCs, as has been suggested by some authors [8, 9]. However, when checking the protein levels of MHC class I (HLA-ABC) and II (HLA-DR), we found only MHC class I expression to be significant increased, while HLA-DR expression remained unchanged (Fig. 1b). This could be a consequence of CIITA, a master gene involved in MHC Class II induction, not translocating into the nucleus, as reported by Tang [10].

By using the software IPA, we clustered these mRNAs with increased expression into common biological pathways and found IFNy signaling and IL-17 signaling to be the first two “top canonical pathways”, leading us to ask how both signaling, together, could contribute to the final immunosuppressive outcome exerted by MSCs. Furthermore, although the mRNAs whose expression increased 2-fold may not be statistically significant, they may result in a potent biological effect when multiple mRNAs belong to the same pathway, since collectively they can potentiate and amplify the pathway. This was also the case for IFNy and IL-17 signaling (SuplFig 2). The role of IFNy in triggering the immunosuppressive properties of MSCs is well recognized by many groups; however, the role of IL-17 in this process remains uncertain. Furthermore, although IL-17 has already been studied as a possible molecule involved in MSCS activation, as far as we know, this is the first study showing the IL-17 pathway as the second most important pathway in this process. Here, we show IL-17 has a crucial role in MSCs activation, as the IL-17 signaling pathway was the second top canonical pathway found by IPA analysis, meaning that, when clustered in pathways, the IL-17 signaling was the pathway with the second most differentially expressed genes among the 311 mRNAs with upregulated expression in MSCs activated by an MLR. This finding highlights the importance of the IL-17 pathway in MSCs immunobiology. Moreover, because we found IL-17RC and Act-1 expression in MSCs and increased levels of them after MSCs activation by an MLR, which agreed with findings that showed IL-17RC to be relatively highly expressed in non-immune cells [11], this result suggests that MSCs might be susceptible and respond to IL-17 signaling, especially if previously activated by cytokines produced by an MLR. Moreover, because MLRs produce IL-17 [12] and we detected, after activation by an MLR, high amounts of IL-6 and IL-8, which are inflammatory cytokines known to be the major cytokines induced by IL-17 signaling (gold-standard cytokines for IL-17 response assessment) (Fig. 2c), these data reinforce our hypothesis that MSCs are activated by IL-17.

Interestingly, IL-17 does not seem to be important for directly triggering the immunosuppressive capabilities of MSCs, as evidenced by the inability of IL-17 to prime MSCs for lymphocyte inhibition. However, because MSCs have strong abilities to induce migration and invasion due to the production of chemokines, such as CXCL8 and CCL8, and production and activation of metalloproteases, such as MMP2 and MMP9, we hypothesized that IL-17 may be important in providing MSCs with intimate contact with lymphocytes, improving lymphocyte sensing of the immunosuppressive molecules produced by MSCs. Meaning that, through chemokine and metalloprotease production, release and activation, MSCs brings lymphocytes closer to MSCs, improving lymphocyte sensing of the immunosuppressive molecules released by the MSCs (alternatively, MSCs could also migrate closer to lymphocytes, producing the same final result). Our findings showing that lymphocytes close to MSCs proliferated less than those that did not migrate through the 8-μm transwell membrane corroborated this idea (Fig. 2f). Furthermore, many previous studies have shown that IL-17 synergizes with other inflammatory cytokines such as IFNy and TNFa to promote the production of IL-6, IL-8, ICAM-1, PGE2 and a range of chemokines [13–16]. The mechanism, at least in part, seems to be dependent on the induction of mRNA stability for these target molecules by IL-17 [17, 18]. Although not tested by our group, it is quite possible that the same process occurs in our model.

Our data show that IL-17 induces high amounts of IL-8 and IL-6 in MSCs, and it is well known that IL-8 is the major chemokine responsible for neutrophil chemoattraction and activation, so the high amount of IL-8 in MSCs treated with IL-17 or the cotreatment leads us to ask how MSCs contribute to immunosuppression through neutrophil modulation. On this topic, Raffaghello et al. showed that IL-6 produced by MSCs dampened respiratory bursts from neutrophils while maintaining unaffected phagocytic activity, matrix adhesion and chemotaxis [19]. Together with Jiang et al., they found this suppression of neutrophil granule release to rescue neutrophils from apoptosis [19, 20], resulting in a decrease in tissue damage, which might contribute to the success of therapy.

The role of IL-17 in MSCs immunobiology has been the subject of a few studies [21–24]. By using different in vitro migration assays, Krstić observed that IL-17 induced migration and invasion in MSCs [24]; however, neither correlations with the immunomodulatory properties of MSCs nor combinations of different inflammatory cytokines were analyzed. In this study, we found that IL-17 treatment alone did not interfere with the immunosuppressive capacity of MSCs. However, Tian found that this treatment inhibited the suppressive capacity of MSCs via mechanisms dependent on the inhibition of Treg expansion [22] while Sivanathan observed the opposite, finding an improved capacity to inhibit lymphocyte proliferation associated with the induction of Tregs [23]. Furthermore, Sivanathan observed that IL-17-treated MSCs were superior modulators of immunological function compared with IFNy-treated MSCs [23]. By treating MSCs with 50 ng/ml IL-17A, the authors were able to inhibit lymphocyte proliferation after PHA stimulation [23]. In contrast, in our hands, treatment of MSCs with 10 ng/ml IL-17 (5 ng/ml IL-17A + 5 ng/ml IL-17F) was not able to inhibit lymphocyte proliferation after anti-CD3/CD28 + IL-2 stimulation. After IL-17 dose titration, we found 10 ng/ml IL-17 to be sufficient for maximal IL-17 signaling activation (evaluated by measuring IL6 and IL8 production) so these discrepant results might be a consequence of the IL-17 dose, especially since Sivanathan used a 10x higher concentration of IL-17A [23]. Furthermore, because PHA stimulates lymphocytes through a still poorly defined mechanism by cross-linking multiple cell-surface glycoproteins [25], we cannot exclude that this potent mitogen may induce a very different signaling network than triggering by anti-CD3/CD28 + IL-2, as already evidenced by the use of concanavalin A [25]. These stimulations might differentially affect the properties of lymphocytes, making them more or less susceptible to MSCs than the physiological pathway activated by anti-CD3/CD28 + IL-2 stimulation. Finally, the use of MSCs derived from the olfactory cells of mice, in contrast to the use of human BM-derived MSCs by us and Sivanathan, might account for the different result observed by Tian [22].

When analyzing cotreatments with inflammatory cytokines, in accordance with our results, Han [21] found IFNy to be sufficient to induce immunosuppression by MSCs and IL-17 to not synergize with IFNy for immunosuppression induction. Interestingly, they observed a synergistic effect of IL-17 when MSCs were treated with IFNy and TNFa, suggesting that the final outcome may depend on the complex set of cytokines available for MSCs activation. Taking into account that pathologies result in an array of inflammatory cytokines, it is possible that IL-17 may contribute to the immunosuppression exerted by MSCs under most inflammatory conditions. In their specific model of liver injury, this effect was due to iNOS induction by IL-17 in IFNy/TNFa-treated MSCs [21].

One of the major concerns regarding the use of MSCs for cellular therapy, especially therapies aimed to induce immunosuppression, is the unwanted possibility of MSCs presenting antigens in very specific situations. Because of this concern, we analyzed the expression of molecules involved in antigen presentation and also performed functional assay. Our studies showed that MSCs treated with IFNy, IL-17 or the cotreatment did not become immunogenic (probably but not exclusively due to the absence of costimulatory molecules), meaning these cells are suitable for cell therapy aimed to induce immunosuppression. In contrast to our finding, the results of Sivanathan showed MHC II and CD40 expression in IFNy-activated MSCs [23]. This discrepant result might be due to the higher amount of IFNy used in their work (500 U/ml IFNy), since, in our study, we used the lowest concentration of IFNy able to stimulate the immunosuppressive capabilities of MSCs without causing any adverse effects.

When looking at the translation of basic knowledge into clinical application, 20 years have passed since the first use of MSCs, but only three phase III randomized studies have proven the effectiveness of MSCs [4] so far. Many discussions has been made in order to analyze why such discrepancies between preclinical and phase III studies, and most of these differences are attribute to differences in MSCs isolation, manipulation and administration [5]. This observation emphasizes the need to learn more about the biology of MSCs to enhance their intrinsic biological activity and thus make better use of these cells. In this way, one of the major discoveries regarding MSCs biology and function came from studies showing that MSCs are activated by inflammatory cytokines, especially IFNy [26], a phenomenon that has become known as “MSCs licensing” or “MSCs priming”. Since then, it has become clear that activated [26] MSCs have much higher therapeutic potential than inactive, steady-state MSCs. Because of this, we hypothesized that the lack of consistent benefit in clinical trials may also be explained by the fact that the injected cells were inactive, steady-state MSCs and therefore their activation was entirely dependent on the patient’s inflammatory status, which varies enormously among patients [27]. Corroborating this idea, preclinical studies have demonstrated the significance of MSCs activated by IFNy with minimal or no adverse effects compared to their inactive counterparts [3, 28], and MSCs from different sources activated by IFNy display gene expression profiles consistent with immunosuppressive potential [29]. In addition to these observations, MSCs activation, despite originally being thought of as a way to improve MSCs efficacy, may be useful to make MSCs preparations more consistent and less heterogeneous among laboratories. Corroborating this supposition, a comparison of phenotypic profiles between inactive and activated MSCs demonstrated that the changes were due to IFNy priming rather than genetic variability [3].

Finally, we speculate that because different pathologies induce different cytokine profiles, it is very likely that the mechanism of MSCs activation differs among diseases, affecting MSCs biological properties of immunoregulation and consequently contributing to the success or failure of MSCs-based therapy. Thus, the use of preactivated MSCs may be useful as a way to improve the biological properties of these cells and minimize variations among MSCs to produce more uniform therapeutic products. However, because MSCs activation seems to be a complex result of many inflammatory cytokines such as IFNy, IL1b, TNFa and IL-17, more studies focused on the use of previously activated cells are needed. These studies can create new possibilities, such as the use of fewer previously activated MSCs for cell therapy or conditioned medium from MSCs. In this way, the goal for future studies should be the identification of the best inflammatory cocktail to activate MSCs to maximize their therapeutic properties while avoiding undesirable expression of molecules involved in antigen presentation. In the not-so-distant future, we envisage a portfolio of MSCs activated by different cocktails specifically designed to target and treat specific diseases.

## Electronic supplementary material

Supplementary figure 1 COX metabolites and chemotactic molecules are involved in the immunosuppression exerted by MSCs. MSCs populations were analyzed after culture in the absence (MSCs) or presence (MSCs t) of MLRs separated by a transwell membrane. A. CXCL8, CCL8 and COX-2 gene expression by real time PCR after 2 and 4 days of incubation. B. Lymphocyte proliferation by CFSE detection (flow cytometry) after 7 days of incubation. Ctrl+ (Positive control, MLR), and test groups: MSCs, MSCs + Indo 10 (Indomethacin 10 μM) and MSCs + Indo 20 (Indomethacin 20 μM). Results are expressed as the mean ± SEM of four independent experiments (B). Experiment A was performed only once. *p < 0,05; ns – not significant. (TIF 205 kb)

High resolution image (PNG 2265 kb)

Supplementary figure 2 Increased canonical pathway activity in MSCs cocultured with an MLR: IFNγ and IL-17 MSCs populations were analyzed after 3 days of culture in the absence (MSCs) or presence (MSCs t) of MLRs separated by a transwell membrane. IFNy an IL-17 signaling pathways provided by Ingenuity Pathway Analysis™ (IPA). The gray shadow represents the molecules whose respective mRNAs were upregulated at least 2-fold after MSCs activation by MLR. Note the mRNAs whose expression increased by more than 5x (marked with ***). (TIF 899 kb)

High resolution image (PNG 2844 kb)

Supplementary figure 3 MSCs cocultured with an MLR proliferate more than steady-state inactive MSCs. MSCs populations were analyzed after 3 days of culture in the absence (MSCs) or presence (MSCs t) of MLRs separated by a transwell membrane. A. Proliferation by cell counts. B. Cell cycle by PI staining (flow cytometry). Results are expressed as the mean ± SEM of twelve independent experiments (A, B). *p < 0,05; ***p < 0,001; ****p < 0,0001. (TIF 137 kb)

High resolution image (PNG 1035 kb)

Supplementary figure 4 IFNy and IL-17 dose definition and the effects of these cytokines on different cellular processes. MSCs populations were analyzed after 3 days of culture in the absence (MSCs)or presence of IFNy (IFNy 10), IL-17 (IL17 10), or both IL-17 and IFNy (10/10). A. Titration of the IFNy and IL-17 dose defined by IL-8 and IL-6 secretion by ELISA and also taking into consideration previously titration [17, 21]. B. MSCs morphology by light microscopy. C. IL-8 and IL-6 secretion by ELISA. D. Titration of the MSCs dose defined by lymphocyte proliferation by CFSE detection (flow cytometry). Ctrl- (Negative control, PBMC without stimulus), Ctrl+ (Positive control, PBMCs + anti-CD3/CD28 + rIL-2), and test groups: Ctrl+ + different concentrations of MSCs (relative to the PBMC responder cells). E. Lymphocyte proliferation by CFSE detection (flow cytometry). Ctrl- (Negative control, PBMC without stimulus), Ctrl+ (Positive control, PBMCs + anti-CD3/CD28 + rIL-2), and test groups: 3,5% MSCs (relative to the PBMC responder cells), IFNy 10 (supernatant of MSCs treated with IFNy), IL17 10 (supernatant of MSCs treated with IL-17), 10/10 (supernatant of MSCs treated with IFNy + IL-17). MSCs were pre-activated for 3 days. Statistical tests were not performed. (TIF 1208 kb)

High resolution image (PNG 3223 kb)

Supplementary table 1 Upregulated mRNAs with a 5-fold change in expression in MSCs cocultured with an MLR. MSCs populations were analyzed after 3 days of culture in the absence (MSCs) or presence (MSCs t) of MLRs separated by a transwell membrane. Results were generated using the Affymetrix Microarrays Platform (PAM). (XLSX 18 kb)
