# Supplementary figures and images for: IL-17 Triggers Invasive and Migratory Properties in Human MSCs, while IFNy Favors their Immunosuppressive Capabilities: Implications for the “Licensing” Process
Source: Stem Cell Rev Rep. 2020 Oct 16;16(6):1266–79. doi: 10.1007/s12015-020-10051-4 (PMC7667142; doi:10.1007/s12015-020-10051-4)

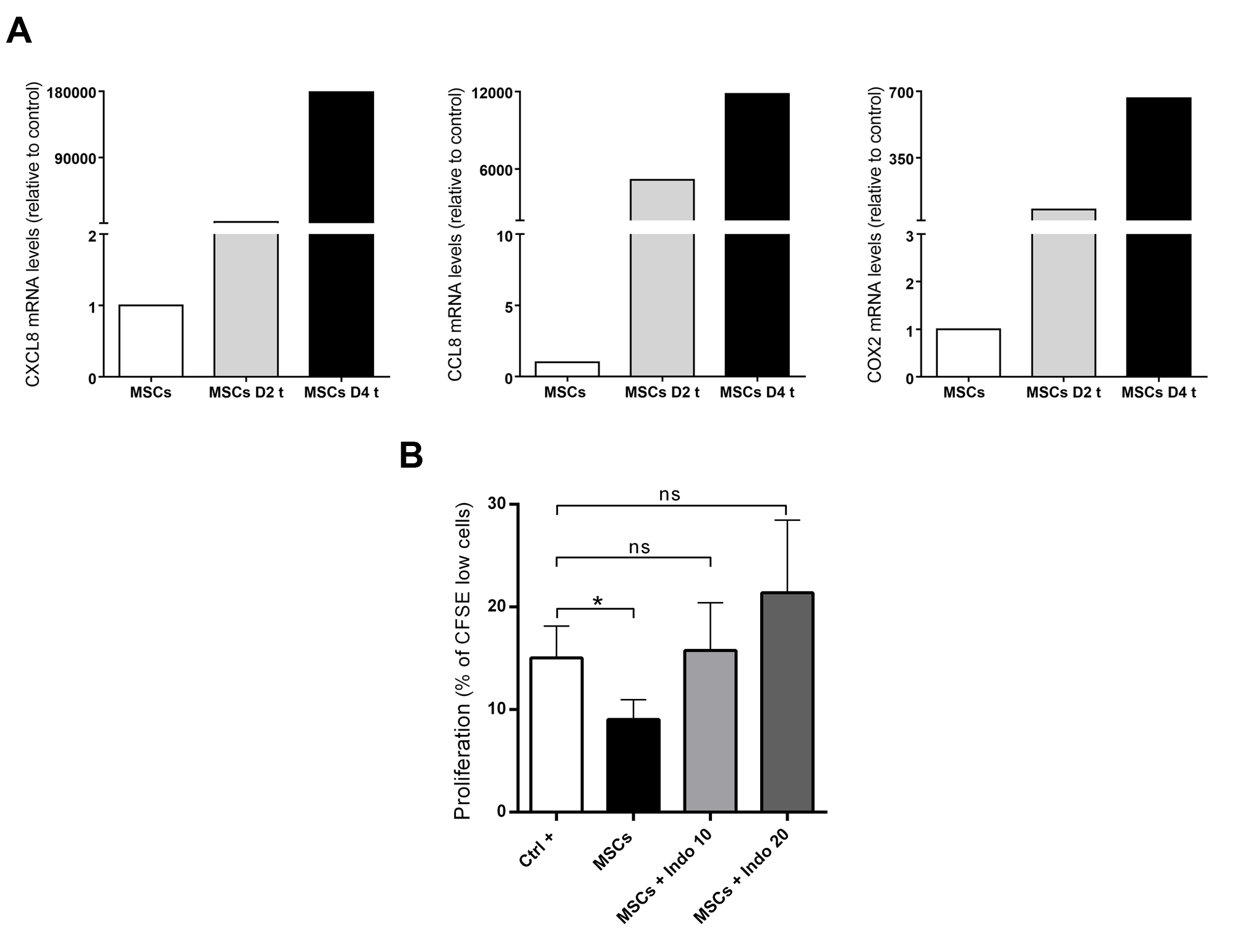

Supplement: Supplementary file 1 — COX metabolites and chemotactic molecules are involved in the immunosuppression exerted by MSCs. MSCs populations were analyzed after culture in the absence (MSCs) or presence (MSCs t) of MLRs separated by a transwell membrane. A. CXCL8, CCL8 and COX-2 gene expression by real time PCR after 2 and 4 days of incubation. B. Lymphocyte proliferation by CFSE detection (flow cytometry) after 7 days of incubation. Ctrl+ (Positive control, MLR), and test groups: MSCs, MSCs + Indo 10 (Indomethacin 10 μM) and MSCs + Indo 20 (Indomethacin 20 μM). Results are expressed as the mean ± SEM of four independent experiments (B). Experiment A was performed only once. *p < 0,05; ns – not significant. (TIF 205 kb) [file 12015_2020_10051_MOESM1_ESM.tif]

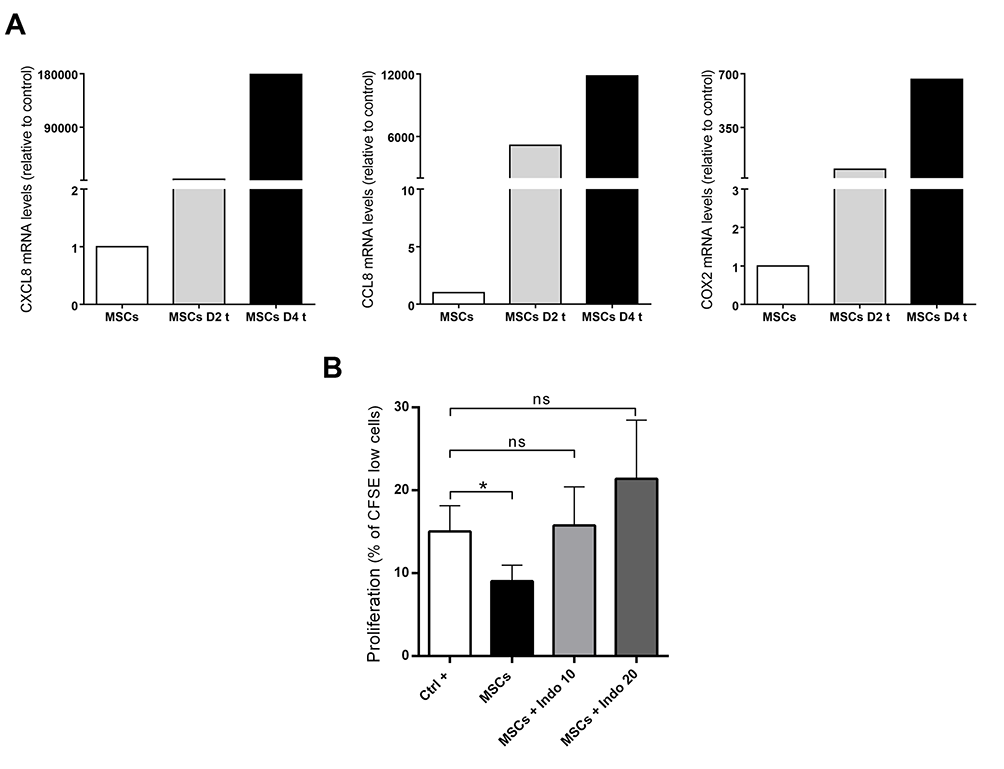

Supplement: Supplementary file 2 — High resolution image (PNG 2265 kb) [file 12015_2020_10051_Fig5_ESM.png]

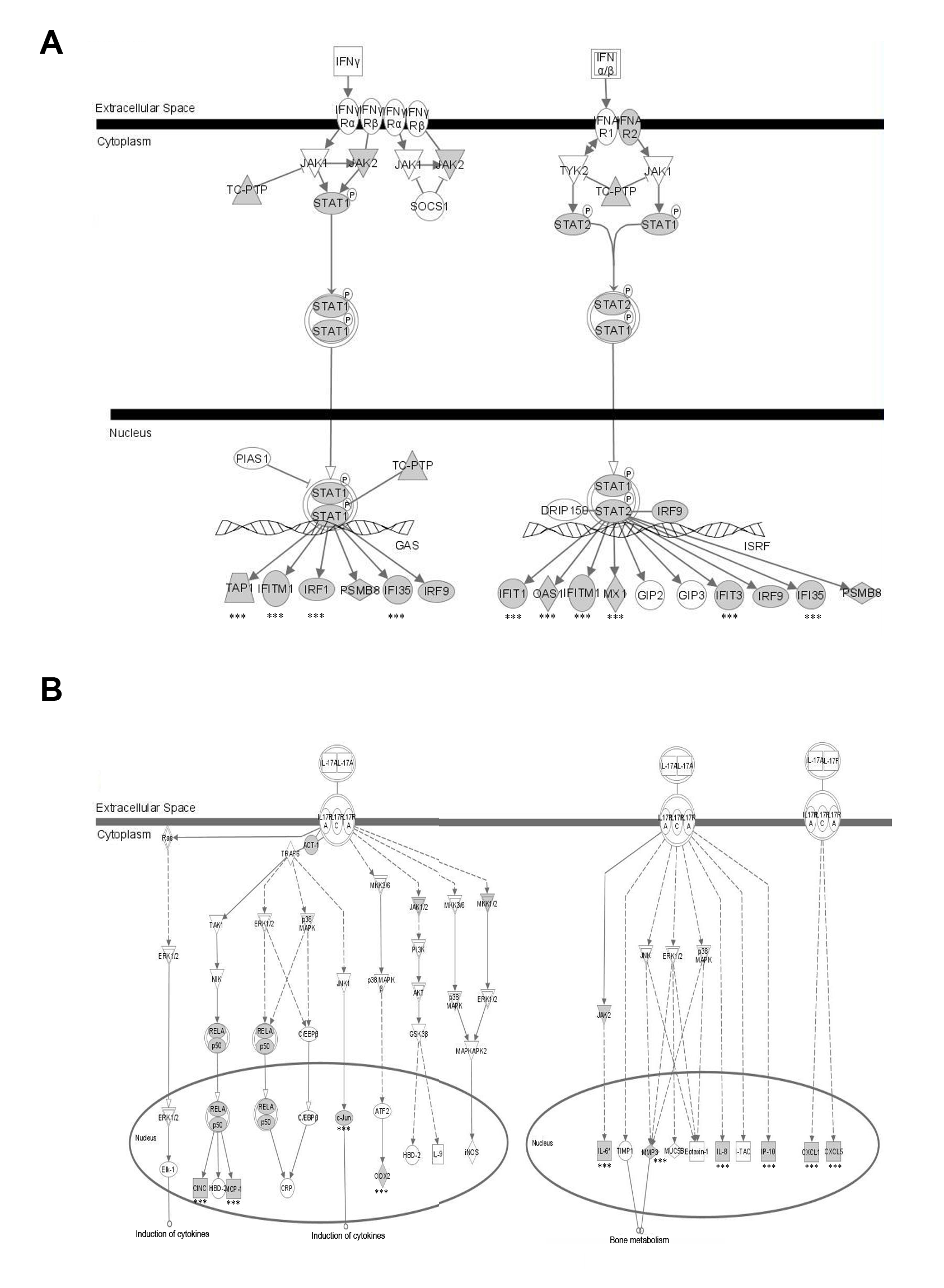

Supplement: Supplementary file 3 — Increased canonical pathway activity in MSCs cocultured with an MLR: IFNγ and IL-17 MSCs populations were analyzed after 3 days of culture in the absence (MSCs) or presence (MSCs t) of MLRs separated by a transwell membrane. IFNy an IL-17 signaling pathways provided by Ingenuity Pathway Analysis™ (IPA). The gray shadow represents the molecules whose respective mRNAs were upregulated at least 2-fold after MSCs activation by MLR. Note the mRNAs whose expression increased by more than 5x (marked with ***). (TIF 899 kb) [file 12015_2020_10051_MOESM2_ESM.tif]

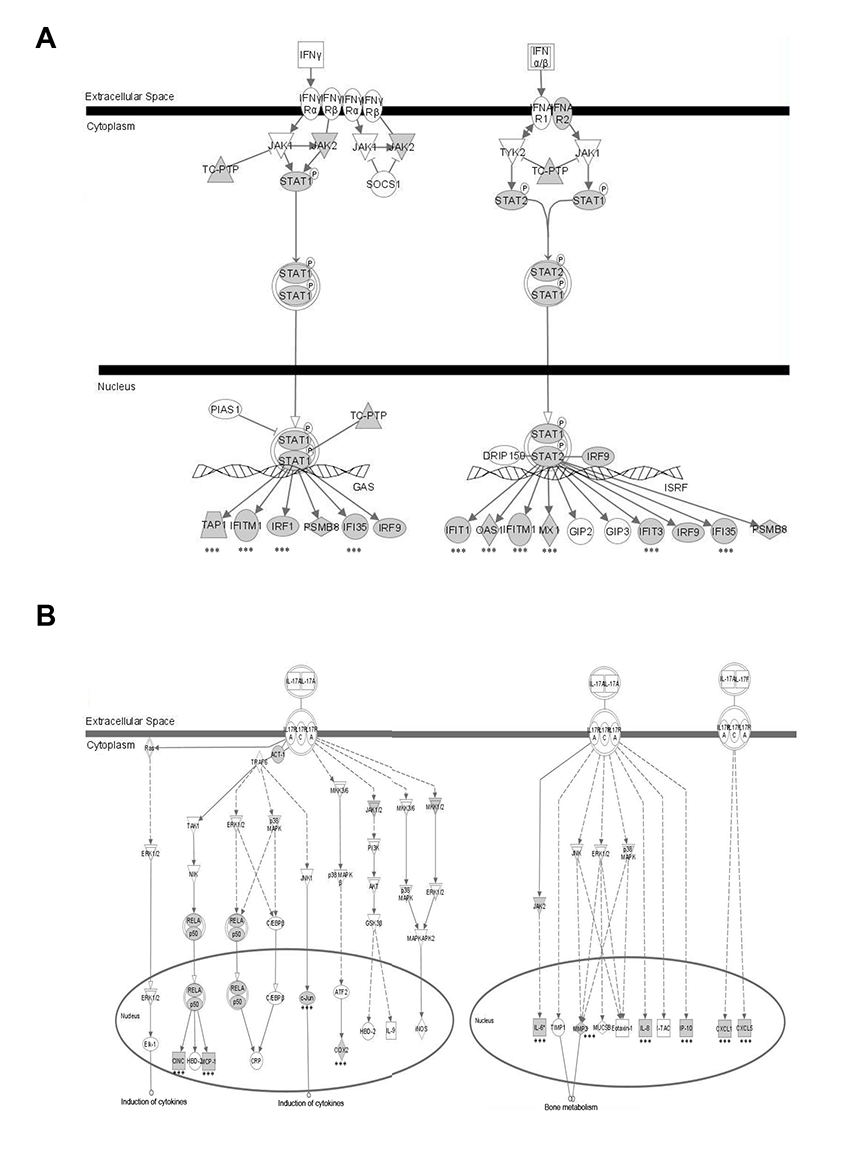

Supplement: Supplementary file 4 — High resolution image (PNG 2844 kb) [file 12015_2020_10051_Fig6_ESM.png]

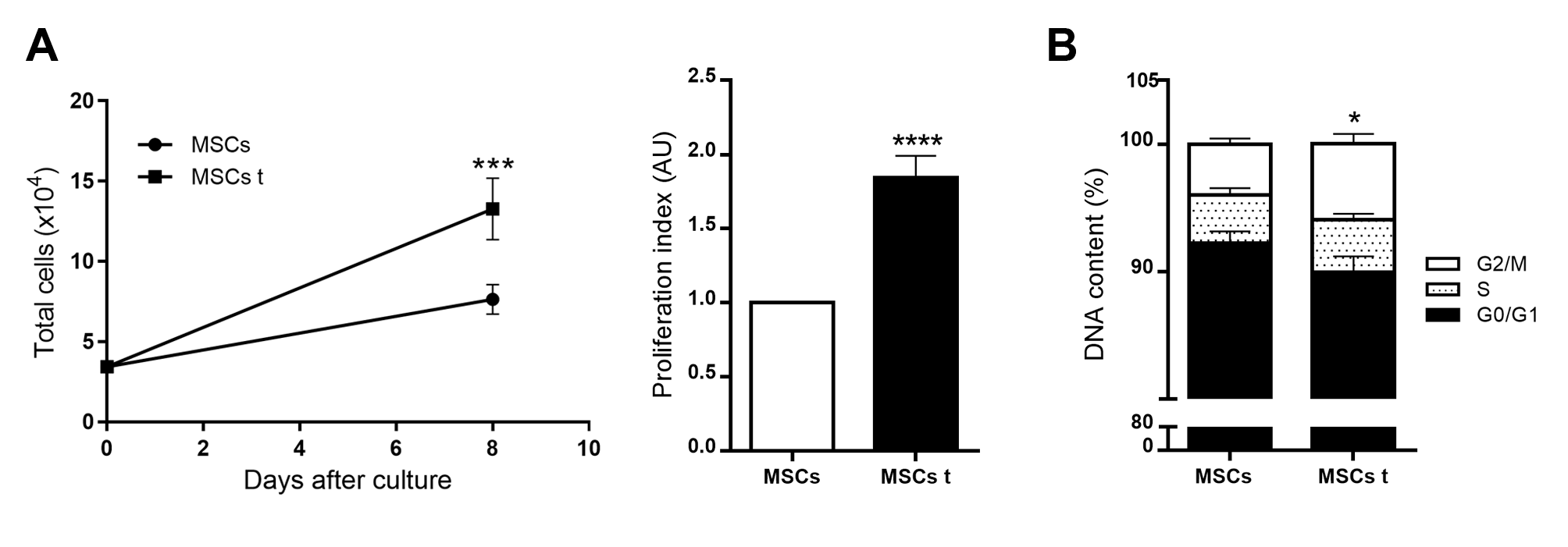

Supplement: Supplementary file 5 — MSCs cocultured with an MLR proliferate more than steady-state inactive MSCs. MSCs populations were analyzed after 3 days of culture in the absence (MSCs) or presence (MSCs t) of MLRs separated by a transwell membrane. A. Proliferation by cell counts. B. Cell cycle by PI staining (flow cytometry). Results are expressed as the mean ± SEM of twelve independent experiments (A, B). *p < 0,05; ***p < 0,001; ****p < 0,0001. (TIF 137 kb) [file 12015_2020_10051_MOESM3_ESM.tif]

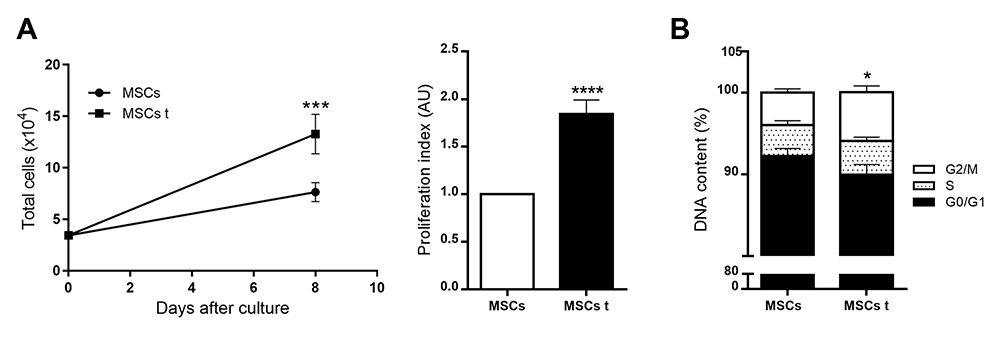

Supplement: Supplementary file 6 — High resolution image (PNG 1035 kb) [file 12015_2020_10051_Fig7_ESM.png]

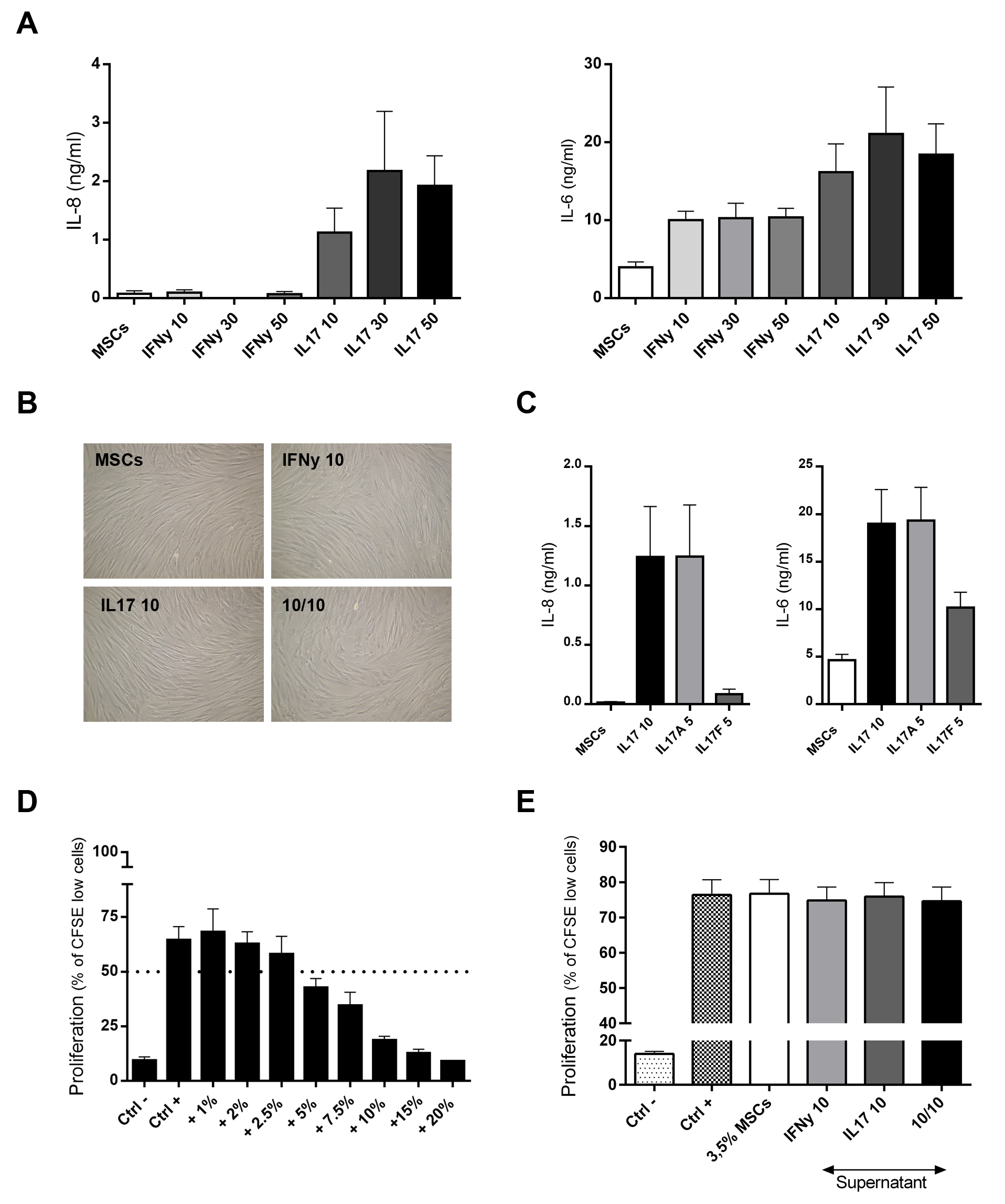

Supplement: Supplementary file 7 — IFNy and IL-17 dose definition and the effects of these cytokines on different cellular processes. MSCs populations were analyzed after 3 days of culture in the absence (MSCs)or presence of IFNy (IFNy 10), IL-17 (IL17 10), or both IL-17 and IFNy (10/10). A. Titration of the IFNy and IL-17 dose defined by IL-8 and IL-6 secretion by ELISA and also taking into consideration previously titration [17, 21]. B. MSCs morphology by light microscopy. C. IL-8 and IL-6 secretion by ELISA. D. Titration of the MSCs dose defined by lymphocyte proliferation by CFSE detection (flow cytometry). Ctrl- (Negative control, PBMC without stimulus), Ctrl+ (Positive control, PBMCs + anti-CD3/CD28 + rIL-2), and test groups: Ctrl+ + different concentrations of MSCs (relative to the PBMC responder cells). E. Lymphocyte proliferation by CFSE detection (flow cytometry). Ctrl- (Negative control, PBMC without stimulus), Ctrl+ (Positive control, PBMCs + anti-CD3/CD28 + rIL-2), and test groups: 3,5% MSCs (relative to the PBMC responder cells), IFNy 10 (supernatant of MSCs treated with IFNy), IL17 10 (supernatant of MSCs treated with IL-17), 10/10 (supernatant of MSCs treated with IFNy + IL-17). MSCs were pre-activated for 3 days. Statistical tests were not performed. (TIF 1208 kb) [file 12015_2020_10051_MOESM4_ESM.tif]

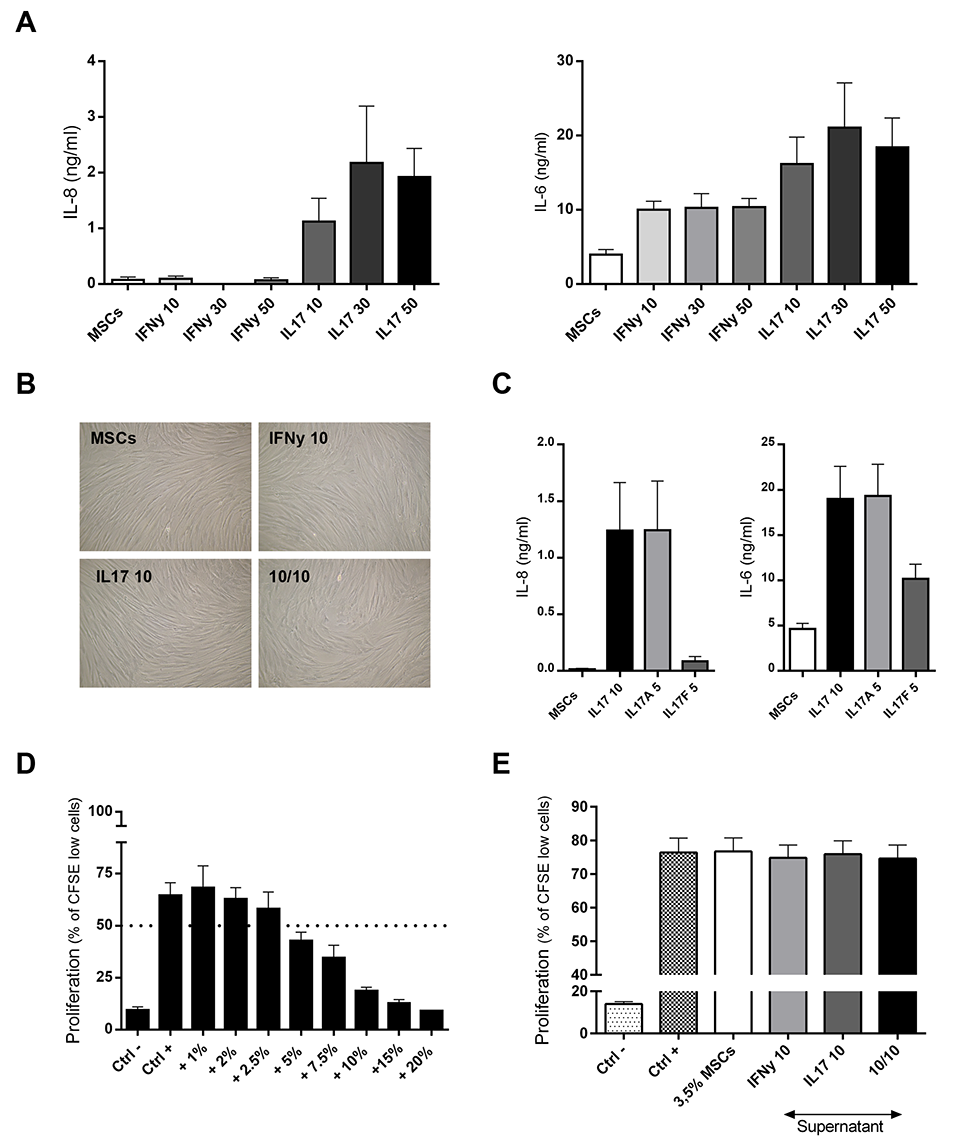

Supplement: Supplementary file 8 — High resolution image (PNG 3223 kb) [file 12015_2020_10051_Fig8_ESM.png]
